# Systematic combinations of major cannabinoid and terpene contents in *Cannabis* flower and patient outcomes: a proof-of-concept assessment of the Vigil Index of Cannabis Chemovars

**DOI:** 10.1186/s42238-022-00170-9

**Published:** 2023-02-08

**Authors:** Jacob Miguel Vigil, Sarah See Stith, Franco Brockelman, Keenan Keeling, Branden Hall

**Affiliations:** 1 Department of Psychology, University of New Mexico, Albuquerque, USA; 2 Department of Economics, University of New Mexico, Albuquerque, USA; 3 MoreBetter, Ltd, Hyattsville, USA

**Keywords:** Cannabis, Health outcomes, Chemovars, Terpenes, Entourage effect, Cannabidiol, Tetrahydrocannabinol

## Abstract

**Background:**

Little is known about the frequency with which different combinations of phytochemicals (chemovars) arise in *Cannabis* flower or whether common chemovars are associated with distinct pharmacodynamics and patient health outcomes. This study created a clinically relevant, user-friendly, scalable chemovar indexing system summarizing primary cannabinoid and terpene contents and tested whether the most frequently consumed chemovars differ in their treatment effectiveness and experienced side effects.

**Methods:**

Between 09/10/2016 and 03/11/2021, 204 people used the freely available, educational mobile software application, Releaf App, to record 6309 real-time consumption sessions using 633 distinct *Cannabis* flower products, unique at the user level, with terpene and cannabinoid potency information. The indexing system is based on retrospective data analysis of the products’ primary and secondary terpene contents and tetrahydrocannabinol (THC) and cannabidiol (CBD) potencies and yielded a total of 478 distinct chemovars. Analyses of covariances (ANCOVAs) were used to compare symptom levels and side effects experienced across the five most common chemovars before and after cannabis consumption for app users overall and for those treating chronic pain and depression or anxiety.

**Results:**

Examination of the five most frequently consumed chemovars showed significant differences in symptom treatment effectiveness for chronic pain and for depression and anxiety (ps < .001). While the effects varied in magnitude, the five chemovars were effective across conditions except for MC61 (mercene .01–0.49%/beta-caryophyllene .01 to 0.49%/THC 20–25%/CBD 0.01–1.0%), which exacerbated feelings of anxiety or depression. The chemovars also differed in their association with experiencing positive, negative, and context-specific side effects, with two chemovars, MC61 and MC62 (mercene .01–0.49%/beta-caryophyllene .01–0.49%/THC 20–25%/CBD 1–5%), generating two to three fewer positive side effects and as much as one more negative and two more context-specific side effects than the other three chemovars.

**Conclusions:**

The findings provide “proof-of-concept” that a simple, yet comprehensive chemovar indexing system can be used to identify systematic differences in clinically relevant patient health outcomes and other common experiences across *Cannabis* flower products, irrespective of the product’s commercial or strain name. This study was limited by self-selection into cannabis and app use and a lack of user-specific information. Further research using this chemovar indexing system should assess how distinct combinations of phytochemicals interact with user-level characteristics to produce general and individualized *Cannabis* consumption experiences and health outcomes, ideally using randomized methods to assess differences in effects across chemovars.

**Supplementary Information:**

The online version contains supplementary material available at 10.1186/s42238-022-00170-9.

*Cannabis* plant strain names, while often a major factor in patient purchasing decisions, have very little scientific or practical relevance. For example, because strains are not usually tested for any specific phytochemical profile, it is not uncommon for producers and retailers to invent original strain names, use secondary sources (e.g., “Leafly”) to reference popular strain names, or even change the name of the strain if sales are not adequate. Unsurprisingly, formal analyses have shown that common, commercially available *Cannabis* plant products are often described with hundreds of strain names in the United States (US), resulting in a false sense of reliability of product contents (Reimann-Philipp et al. 2020). In spite of this limitation, *Cannabis* remains among the most versatile medicinal plants ever discovered (Russo 2007; Stith et al. 2018) and is increasingly becoming a primary or secondary form of medication for tens of millions of people in the US. The *Cannabis* plant can contain over one hundred phytocannabinoids, and several hundreds of terpenes, terpenoids, and other phytochemicals with known pharmaceutical relevance (McPartland 2012; Fischedick 2015), not including those that have yet to be identified, resulting in a nearly infinite number of possible chemical combinations, often referred to as “chemovars” with differing pharmacodynamics and potential applications (Andre et al. 2016; Lewis et al. 2018; Aviram et al. 2021; Stith et al. 2018; Vigil et al. 2020; Vigil et al. 2017).

Previous studies that have looked into the medicinal potential of *Cannabis* have mainly analyzed the two most well-known phytocannabinoids: tetrahydrocannabinol (THC) and cannabidiol (CBD) (National 2017; Stith et al. 2019). Very little research has attempted to assess how heterogeneous combinations of other naturally occurring chemical constituents within the *Cannabis* plant, such as common terpenes, may be more or less effective for treating various health symptoms. What is known about the potential medicinal benefits and toxicity of terpenes is primarily derived from work on other plants and their essential oils, where often the terpenes and terpenoids are found in much higher concentrations than can naturally occur in the *Cannabis* plant (Andre et al. 2016; Lorenzetti et al. 1991; Falk et al. 1990). These studies show that monoterpenes such as alpha-pinene, myrcene, and terpinolene have both pharmaceutical and industrial applications (Behr and Johnen 2009; Surendran et al. 2021; Ito and Ito 2013; Menezes et al. 2021). Though the combined effects of cannabinoids and terpenes are often hypothesized to treat numerous health conditions, (McPartland 2012; Ferber et al. 2019; Russo et al. 2005; Russo and Guy 2006; Kamal et al. 2018) we are unaware of any empirical study directly contrasting patient outcomes from exposure to different kinds of “entourage effects,” i.e., the commonly assumed synergistic and therapeutic potential from simultaneously consuming multiple phytochemicals from the *Cannabis* plant. Currently, one of the most critical barriers to advancements in the medical use of cannabis is the lack of a coherent classification system, so that naturally heterogenous *Cannabis* plants can be reliably categorized according to their unique phytochemical profiles and applications (National 2017; Stith and Vigil 2016).

The goal of the present report is to introduce a comprehensive and user-friendly *Cannabis* plant classification system that can be easily referenced by scientists, health providers, and patients for identifying basic chemotypic properties of plants, regardless of a product’s arbitrary strain name. We use a large database of real-time cannabis administration sessions to create a fluid nomenclature system for indexing *Cannabis* flower strains based on the plant’s primary and secondary terpene concentrations and absolute THC and CBD potency levels. We then conduct a “proof-of-concept” analysis by contrasting the most frequently consumed plant chemovars for any potential differences in their associations with patient symptom relief and side effect experiences. We focus on patient symptom relief associated with chronic pain, depression, and anxiety, due to the high prevalence of these conditions in medical cannabis patient registries and in the general US population.

## Methods

### Study design

The study design was reviewed and deemed exempt from further oversight by the Institutional Review Board at the University of New Mexico due to the retrospective and anonymized nature of the data. The owner of the Releaf App^™^, MoreBetter, Ltd., provided de-identified data to the investigators subject to a data use agreement. The freely available Releaf App^™^ educational mobile software was designed to enable users to document the labeled characteristics of their cannabis products, cannabis usage characteristics (e.g., dosing and route of administration), user health conditions, baseline and momentary symptom intensity levels, and experienced side effects during real-time, in vivo, self-administration sessions. State-legal cannabis product labels are required to include THC and CBD levels. Validation of these levels occurs through state-authorized cannabis testing laboratories with some of the labs also providing information on terpene concentrations. The potential terpenes available for entry in the ReleafApp™ software include alpha-pinene, beta-pinene, beta-caryophyllene, caryophyllene-oxide, alpha-humulene, linalool, limonene, myrcene, ocimene, terpinolene, terpineol, alpha-phellandrene, alpha-terpinene, fenchol, camphene, valencene, garaniol, guaiol, alphabisabolol, and farnasene. The Releaf App^™^ includes 52 health symptoms and 47 possible side-effects. The study sample includes treatment sessions with post-consumption symptom intensity levels reported at least once within the first hour after session initiation.

**Fig. 1:**
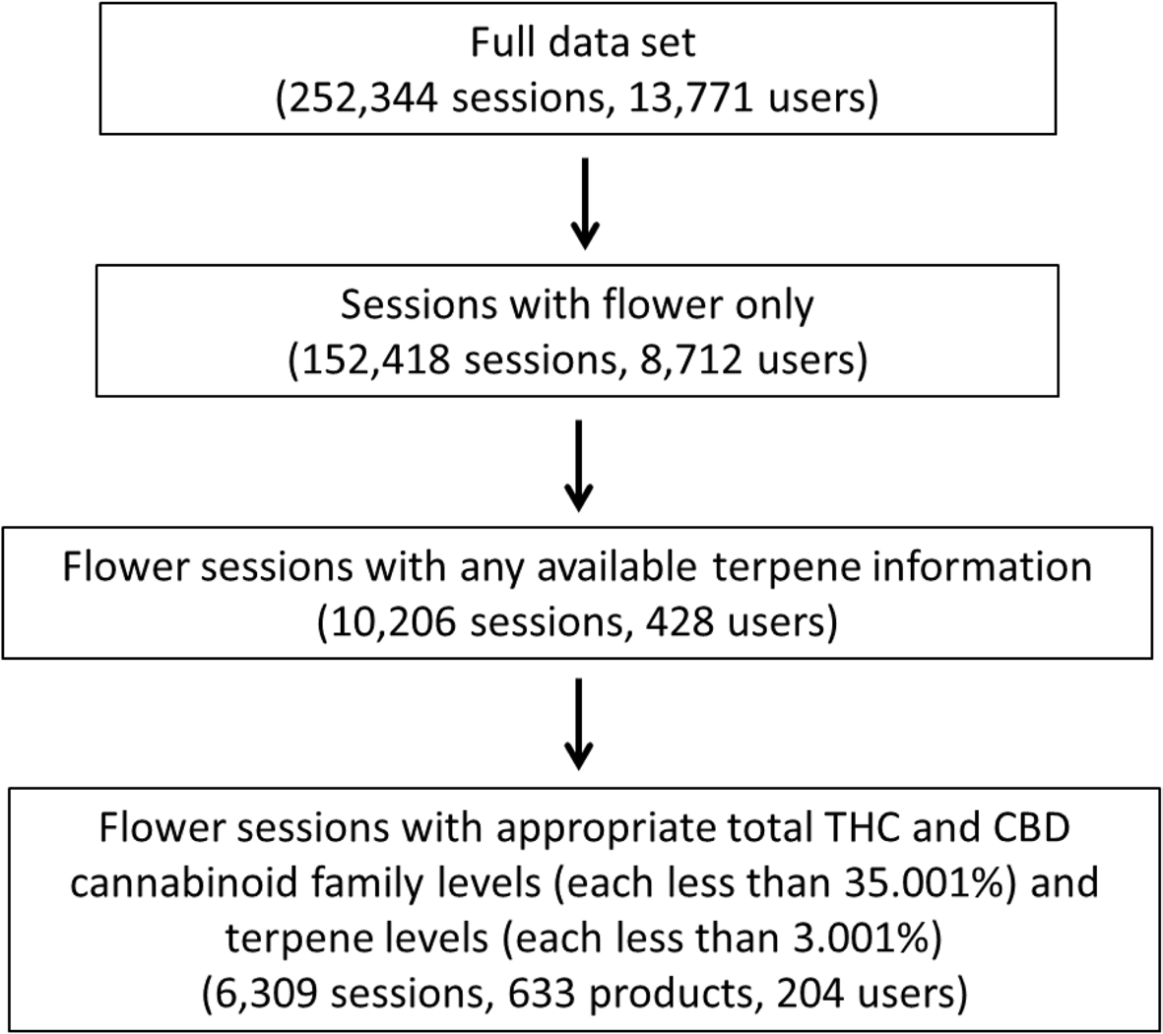
Diagram of flowchart of inclusionary criteria for data analyses

A description of data filtering procedures is shown in Fig. 1. The initial dataset consisted of 252,344 sessions recorded by 13,771 users between June 6, 2016 and March 11, 2021. Only the sessions using flower products (60.4% of total sessions) were included in the dataset, and 6.7% of the flower sessions included laboratory-provided information on the product’s terpene levels. Recorded potency levels for labeled THC, THCa, THCv, and THCva were aggregated (THC family), as were levels of CBD and CBDa (CBD family). To avoid confounding from user entry error, cutoff thresholds for cannabinoids and terpenes were selected based on the biological limitations of the *Cannabis* plant (Reimann-Philipp et al. 2020). The cutoff thresholds for reasonably labeled cannabinoid family levels were set at 35.0%/dry wt., and the cutoff for each of the 20 terpenes was set at 3.0%/dry wt. Sessions reporting levels that were higher than these cutoffs were excluded from the final analyses. Each product is unique at the user level, i.e., if two users were to purchase the same product, it would appear in the data as two separate products.

The final analyzed sample included 204 users who completed 6309 cannabis administration sessions using 633 distinct products with bona fide terpene and cannabinoid content labels between 09/10/2016 and 03/11/2021.

### Chemovar indexing method

In order to accommodate measurement error in conventional laboratory testing results, to allow for natural variations in potency within a product batch, and to enable cannabis users the ability to better manage the nearly limitless possible number of chemovars across products, the absolute potency volumes provided on product labels were categorized on ordinal scales, separately for terpene and cannabinoid contents. To create the index system and the associated treatment variables, distinct plant chemovars were categorized according to a 4-character coding system that broadly describes the relative magnitudes of the primary and secondary terpenes detected and THC and CBD potency levels. The first two characters are the alphabetic symbols for the 20 recorded terpenes, with the highest concentrated terpene in the first place and the terpene with the 2nd highest concentration in the second place. Table 1 shows the number of sessions with information for each of the terpenes, the frequency that information was provided for each terpene, the average recorded concentration volume for each terpene, and the alphabetic index code for the terpenes.

**Table 1:** *Cannabis* plant terpene alphabetic codes and usage characteristics

Terpene (Index Code)	N terpene session recordings	Frequency of terpene recordings	Average volume (SD)
Myrcene (M)	5668	89.8%	.506 (.485)
Beta-caryophyllene (C)	5272	83.6%	.294 (.318)
Limonene (L)	4961	78.6%	.310 (.356)
Alpha-pinene (A)	3985	63.2%	.171 (.277)
Alpha-humulene (H)	3.919	62.1%	.125 (.210)
Linalool (N)	3495	55.4%	.160 (.261)
Beta-pinene (B)	3678	58.3%	.135 (.269)
Terpinolene (T)	2203	34.9%	.412 (.532)
Ocimene (O)	776	12.3%	.298 (.462)
Alphabisabolol (I)	914	14.5%	.041 (.036)
Caryophyllene-oxide (R)	679	10.8%	.336 (.550)
Garaniol (G)	515	8.2%	.111 (.081)
Camphene (E)	134	2.1%	.135 (.282)
Guaiol (U)	171	2.7%	.094 (.195)
Alpha-terpinene (J)	79	1.3%	.524 (.994)
Terpineol (P)	133	2.1%	.135 (.362)
Fenchol (F)	71	1.1%	.326 (.762)
Valencene (V)	54	0.9%	.103 (.164)
Alpha-phellandrene (D)	5	0.1%	.247 (.181)
Farnasene (S)	0	0.0%	--

All terpenes available for selection in the ReleafApp™ are included

Within the coding system itself, the relative magnitudes of the terpene concentrations are indicated with the presence of superscript(s) “^+^” following the alphabetic symbol indicating one of 4 possible concentration levels: (no superscript) = 0.01 to 0.49%/dry wt.; “^+^” = 0.50–0.99%/dry wt.; “^++^” = 1.00–1.99%/dry wt.; and “^+++^” = 2.00–3.00%/dry wt. In the main coding system, the “-” indicates the absence of a 2nd identified terpene and a “/” between terpenes in the 1st and 2nd places indicate exactly matched concentration levels.

The third and fourth places in the coding system are reserved for the plant’s absolute THC and CBD potency levels, respectively. The units consist of digits across two separate scales (1–8 for THC, and 0–8 for CBD) representing the distribution of the most common cannabinoid levels listed on product labels. The possible THC codes (the 3rd place in the index code) are as follows: 1 = 0.01–0.9%; 2 = 1–4.9%; 3 = 5–9.9%; 4 = 10–14.9%; 5 = 15–19.9%; 6 = 20–24.9%; 7 = 25–29.9%; 8 = 30–35%. (Given the scarcity of *Cannabis* flower strains with no [0.0%] detectable THC, the value “0” is not used for this scale.) The possible CBD codes (the 4th place in the index code) are as follows: 0 = 0.0%; 1 = 0.01–0.9%; 2 = 1–4.9%; 3 = 5–9.9%; 4 = 10–14.9%; 5 = 15–19.9%; 6 = 20–24.9%; 7 = 25–29.9%; 8 = 30–35%.

As shown in Supplemental Table S1, a total of 478 unique chemovar codes were identified in the current sample. The five most frequent chemovar index codes are described in Table 2. As shown in Table 2, four of the five most frequently represented chemovars contained mercene as the primary or secondary terpene, all the chemovars had THC levels that ranged between 15 and 25%, and all but one had less than 1% CBD. Although the exemplar chemovars were comprised of products with differing strain names, the product labels showed strong trends indicating either a *Cannabis sativa* or *Cannabis indica* dominance, or hybridization of the two.

**Table 2:** Descriptions of frequently consumed *Cannabis* flower chemovar index codes

VICC Index Code	N sessions	Primary terpene	Secondary terpene	THC potency	CBD potency	Commercial names	%Sativa/Indica/Hybrid
LM60	231	Limonene (.01–.50%)	Mercene (.01–.50%)	20–25%	0%	Grapefruit Durban, 24K Gold	80/4/16
M^+^A50	212	Mercene (.50−1.0%)	Alpha-pinene (.01–.50%)	15–20%	0%	9 Pound Hammer, Blueberry Cookies	0/28/72
MC61	223	Mercene (.01–.50%)	Beta-caryophyllene (.01–.50%)	20–25%	.01–1.0%	Starfall, Scarlet Queen	0/1/99
MC62	211	Mercene (.01–.50%)	Beta-caryophyllene (.01–.50%)	20–25%	1%-5%	Royal Purple Kush	0/100/0
T^+^L60	222	Terpinolene (.50–1.0%)	Limonene (.01–.50%)	20–25%	0%	Cookies and Cream, Florida Black Haze #14	79/0/21

The VICC (Vigil Index of Cannabis Chemovars) uses a 4-unit coding system to indicate relative primary terpene and cannabinoid concentrations. Concentrations represent approximations with the tenths place rounded up to the next whole digit. Valid percentages of sativa/indica/hybrid label descriptions are shown

### Study outcomes

The study objectives are to identify common examples of unique chemovars and evaluate whether differences exist in their effectiveness at reducing the severity of patients’ symptoms, and their associations with experienced side effects. Symptom relief is measured by subtracting the (post-dosing) lowest recorded symptom intensity level from the baseline (pre-dosing) intensity level, resulting in potential symptom changes ranging between − 10 (maximum symptom relief) and 9 (minimum symptom relief/maximum increase in symptom severity) points. (Only sessions with starting symptom intensity levels of one or more are included, so as to include only sessions attempting to treat a measurable health symptom.) The 47 possible side effects are categorized into 17 negative side effects, 19 positive side effects, and 11 context-specific side effects. We convert these categories of side effects into continuous variables measuring the absolute number of total side effects in each category that the user selected. The full list of possible side effects, the frequency in which they were reported, and their categorical distinctions are shown in Supplemental Table S2. The most commonly reported negative side effects in the current sample are dry mouth (40.6% of sessions) and red eye (26.8%), the most common positive side effects are feeling chill (63.1%) and relaxed (56.2%), and the most common context-specific side effects are feeling high (56.3%) and tingly (33.0%).

### Statistical analysis

Analyses of covariances (ANCOVAs) were used to measure the relationships between the exemplified chemovars and symptom relief within the first hour following consumption and between the chemovars and experienced side effects reported during that first hour. Baseline symptom intensity level was included as a covariate given the relationship between the starting symptom level and the magnitude of potential symptom relief (Stith et al. 2018; Stith et al. 2019). We also included the product’s total terpene contents as a covariate to control for the volume of additional terpenes not represented in the primary or secondary indexing position, and we included the total number of side effects recorded as a covariate for examining each side effect category. The analyses focused on the full sample, as well as two patient subgroups: (a) consumption sessions used to treat pain (*n* = 2372, 37.6% of total sample) and (b) consumption sessions for treating either anxiety symptoms or depression (*n* = 1,062, 16.8%). We group these conditions together because they often occur concomitantly and to maintain a large enough sample for analysis. In order to ensure that the results are not driven by users with disproportionate numbers of session entries, robustness checks were conducted limiting the analyses to products that were tested within the first ten sessions recorded by a user. Analyses were conducted using IBM SPSS Statistics 23 (IBM 2015).

## Results

**Fig. 2:**
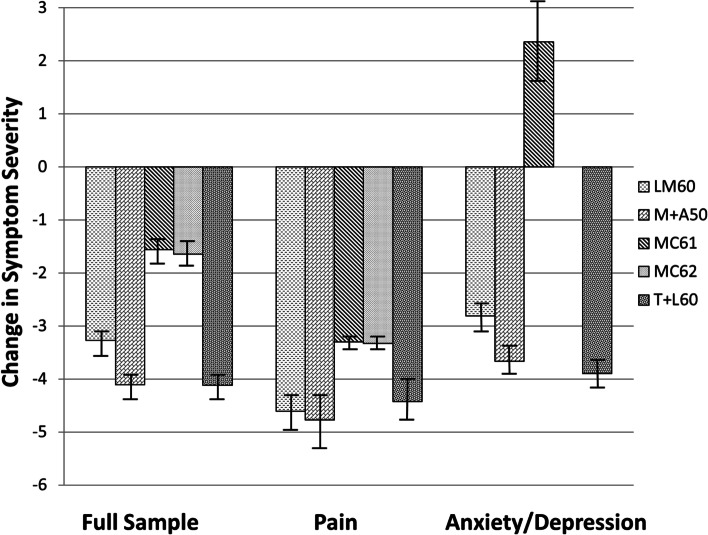
Estimated mean changes in symptom severity for distinct chemovars. Note: The estimated means are adjusted for baseline symptom severity level and total volume of terpenes in each product (bars indicate 95% confidence intervals). The MC62 variant was not represented in the analyses for anxiety/depression due to small sample sizes

ANCOVAs were used to examine any group differences in patient symptom relief and side effects experienced across the chemovars. In analyzing symptom relief, baseline symptom intensity level and total terpene contents were included as covariates for analyses run separately for the overall sample, for patients treating pain, and for patients treating anxiety/depression. Significant group differences in symptom relief were found across the chemovars in the total sample, *F*(4,1072) = 52.28, *p* < 0.001, as well as for patients treating only pain, *F*(4,480) = 12.74,* p* < 0.001, and for patients treating anxiety or depression, *F*(3,185) = 67.26, *p* < 0.001. Figure 2 shows the estimated marginal means for symptom relief for each of the exemplar chemovar index codes. The results suggest that exemplar chemovars with any discernable amounts of CBD provide less symptom relief than those without CBD. Additionally, the variants with slightly higher-than-average levels (between 0.50 and 1.0%) of mercene and terpinolene appeared to be associated with reliably stronger therapeutic effects.

**Fig. 3:**
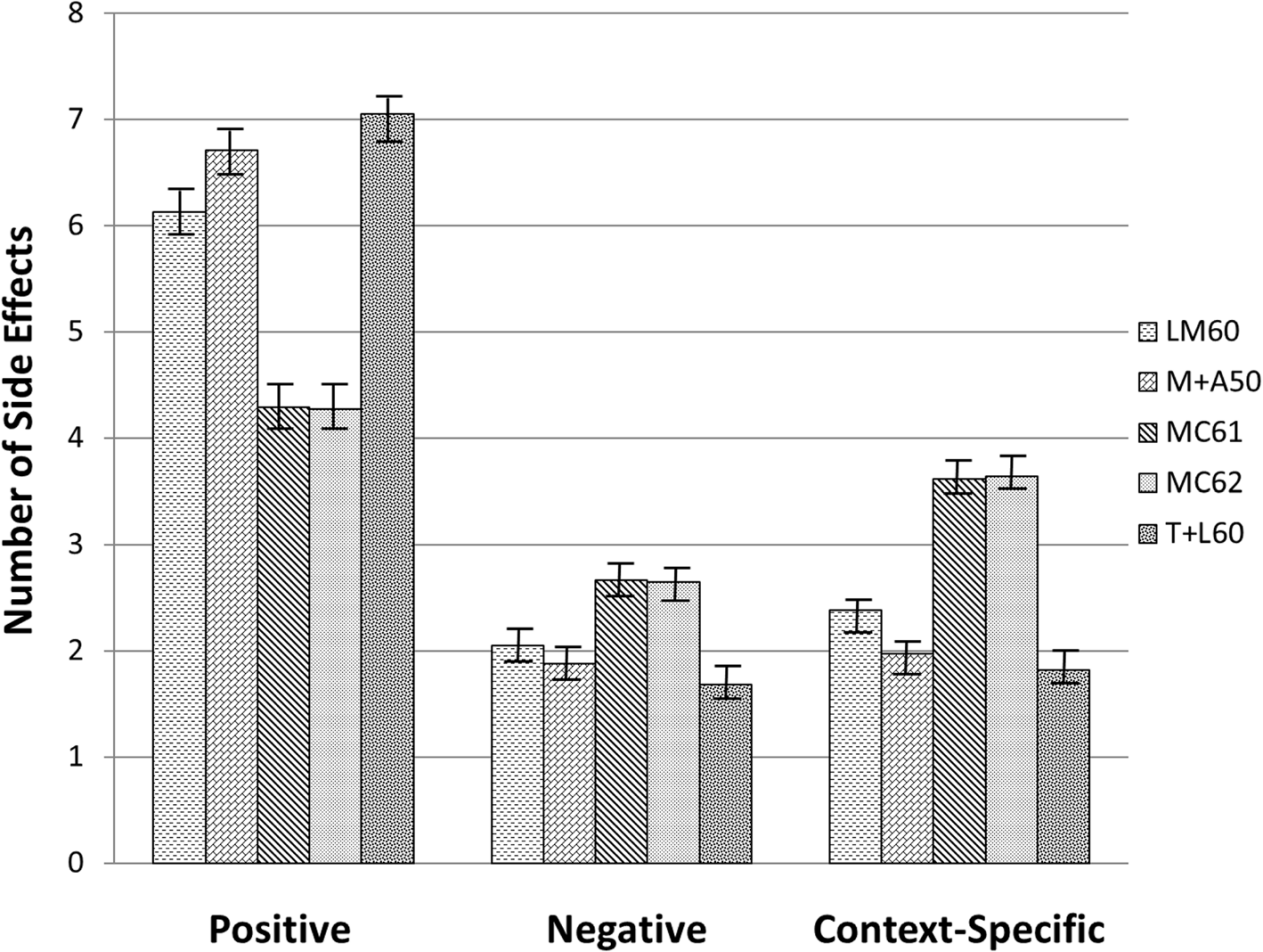
Estimated number of side effects for distinct chemovars. Note: The estimated means are adjusted for baseline symptom severity level, total volume of terpenes in each product, and total number of recorded side effects (bars indicate 95% confidence intervals).

ANCOVAs were then used to examine the total number of positive, negative, and context-specific side effects for each of the exemplar chemovars, while controlling for baseline symptom level, total terpene contents, and total number of side effects recorded. Using the entire sample, significant differences were found in the frequency of experiencing positive side effects, *F*(4,1050) = 73.73, *p* < .001; negative side effects, *F*(4,1050) = 11.28, *p *< .001, and context-specific side effects *F*(4,1050) = 68.89, *p *< .001. Figure 3 shows the estimated marginal means for each of the three side effect categories for each chemovar. The results suggest exemplar chemovars with slightly higher-than-average levels of mercene and terpinolene (between 0.50 and 1.0%) and no discernable volume of CBD appeared to be associated with the greatest likelihood of experiencing positive side effects and the least likelihood of experiencing negative or context-specific side effects, whereas variants with the lowest terpene levels and any detectable amounts of CBD were associated with the least likelihood of experiencing positive side effects and the greatest likelihood of experiencing negative or context-specific side effects.

Finally, robustness checks controlling for number of product entries were conducted by limiting analyses to specific products that were consumed within the first 10 sessions using the full patient sample. ANCOVAs again revealed significant differences across the chemovars for symptom relief, *F*(4,218) = 8.17, *p *< .001, the occurrence of positive side effects, *F*(4,215) = 18.02, *p* < .001; and to a lesser extent negative side effects, *F*(4,215) = 11.09, *p *< .001; and context-specific side effects, *F*(4,215) = 15.37, *p *< .001. Chemovars with the higher levels of mercene and terpinolene (i.e., M^+^A50 and T^+^L60) and no detectable volume of CBD were again associated with the greatest symptom relief (estimated mean changes in symptom intensity = −3.08 and − 3.66, SEs = .22 and .23, respectively), a higher likelihood of experiencing positive side effects (estimated mean number of positive effects = 6.89 and 8.03, SEs = .25 and .27), and the lowest likelihood of experiencing negative side effects (estimated means = 1.87 and 1.43, SEs = .18 and .20) and context-specific effects (estimated means = 2.27 and 1.56, SEs = .15 and.17). In contrast, the chemovars with any detectable levels of CBD (i.e., MC61 and MC62) were again associated with the least relief (estimated mean changes = −1.53 and − 1.55, SEs = .30 and .50), along with the fewest positive side effect experiences (estimate means = 4.46 and 4.00, SEs = .35 and .56), the most negative side effect experiences (estimate means = 3.56 and 2.75, SEs = .25 and .40), and the most context-specific side effect experiences (estimate means = 3.00 and 4.25, SEs = .21 and .34).

## Discussion

The present report introduces a common-sense and user-friendly, yet comprehensive and generative indexing system for categorizing *Cannabis* flower products based on the information commonly reported on product labels throughout the US. Legal cannabis markets in the US require product labels to include THC and CBD potency levels, and many state-licensed testing laboratories also provide primary terpene analyses (Ibrahim et al. 2019). Although decades have passed since California first legalized medical cannabis in 1996, no systematic approach exists for directing patients towards the cannabis products most likely to improve their symptoms. In dispensaries nationwide, patients are directed towards products based on informal, scientifically arbitrary strain names—designations which cannot be measurably related to any underlying plant characteristics. While previous research has successfully identified some broad distinctions in the terpene profiles that vary across *C. sativa* and *C. indica* (or hybrid) plant strains (Hazekamp et al. 2016), these basic categories do not offer specific information on the unique characteristics of a product’s constituents. Therefore, a patient cannot reliably know, based on the current product descriptions, whether one type of flower will have a similar effect to another or whether even products labeled as being the same strain will reliably generate the same effect. Herein we assign each heterogeneous *Cannabis* flower product with a unique indexing code describing the relative magnitudes of the product’s primary and secondary terpene contents and the product’s absolute THC and CBD potency levels, using commonly available, easily testable, and empirically comparable phytochemical measurements. The indexing system is validated as showing statistically and clinically significant differences in reported symptom relief for specific health conditions and differences in the side effects experienced across the modeled chemovars. By using information readily available on legal, retail cannabis product labels, beyond current nominal methods of categorization and extending beyond THC and CBD potency levels, the system enables clinicians, patients, and scientists to better customize and target *Cannabis* flower products for specific indications. Using the current dataset of 633 products, we were able to observe 478 unique chemovars. However, as electronic data recording systems, such as the Releaf App™, become increasingly populated and growers intentionally and unintentionally continue to hybridize plants, several hundred additional chemovars will likely be identifiable within the next few years.

The second aim of the current report was to test the research hypothesis that the distinct plant chemovars represented by our indexing system differ in their ability to treat health conditions. In addition to validating the indexing system, this study is among the first to test for entourage effects from distinct plant variants on patient outcomes or even that varying naturally occurring *Cannabis* plant terpene levels differentially affects patient outcomes. While we only contrasted the effects of five distinct chemovars, they provided a proof-of-principle that the indexing system can be referenced against consumer-generated databases to predict differences in the effectiveness and experienced side effects from varying plant strains. Specifically, symptom relief was greatest after consumption of plant variants with slightly higher than average levels of the terpenes, mercene, and terpinolene (e.g., M^+^A50 and T^+^L60) and non-detectable levels of CBD. In contrast, chemovars with any detectable levels of CBD (e.g., MC61 and MC62) provided the least relief, the fewest positive side effects, and the most negative and context-specific side effects. These findings are consistent with previous research showing that naturally abundant CBD in *Cannabis* flower may act as an inhibitor of optimal treatment for certain health conditions such as gastrointestinal pain (Stith et al. 2019; Li et al. 2019). Likewise, the utility of mercene for numerous pharmaceutical applications (e.g., anxiolytic, antioxidant, anti-aging, anti-inflammatory, analgesic) is well established, (Behr and Johnen 2009; Surendran et al. 2021) and though less researched, terpinolene has also been shown to have overlapping (e.g., antioxidant) and unique (e.g., sedative) therapeutic applications (Ito and Ito 2013; Menezes et al. 2021).

The proposed cataloging system and the large database from which the individual indices and their associated effects are based are not without limitations. While having the potential to help guide future randomized controlled trials (RCTs) on the pharmacodynamics of specific *Cannabis* plant chemovars, the current study is limited by its observational nature and lack of a control group. Although the Releaf App™ dataset is among the largest collections of real-time cannabis usage sessions in the US, it is limited by the information users have available to enter into the app. Presently, relatively few cannabis-based products offer full descriptions of their terpene concentrations, as these tests add to operational expenditures and are rarely required by state level regulatory agencies. The incompleteness of the dataset is particularly constraining when the chemovars are analyzed at the most granular level, using the separate indices shown in Supplemental Table 1. However, the system can be flexibly adapted to include overlapping properties (e.g., terpene volumes that are close in order and/or magnitude) and broader product distinctions. The current dataset is also limited by its reliance on self-selection and self-reporting. It is possible that users may be excluded from the dataset if they choose to no longer use the app because, for example, they are either satisfied or dissatisfied with their cannabis product choices. Likewise, individual-level information about the cannabis users observed in this study, including their age, gender, geographical location, medical history, history and frequency of medicinal and recreational cannabinoid use, and concurrent medicines and therapies, is missing, and future research is needed to cross-examine the effects of personal characteristics with product-level and usage-level factors. Finally, it is important to note that, while the current coding system congregates the isoforms of THC and the isoforms of CBD together, the isoforms can have differing activities at the major cannabinoid-sensitive receptors (e.g., CB1, CB2, TRPV1, TRPA1, TRPM8) (Andre et al. 2016; Muller et al. 2019; Alves et al. 2020; Lucas et al. 2018). As comprehensive laboratory testing becomes more pervasive, there will eventually be enough data to create a more sophisticated indexing system that incorporates more detailed chemotypic factors than is currently allowed.

In conclusion, the index system described herein enables healthcare providers, patients, scientists, and cannabis retailers to easily categorize *Cannabis* products based on measurable plant characteristics beyond THC and CBD in ways that systematically relate to differing levels of symptom relief and side effect reporting.

## Supplementary Information

**Additional file 1:** **Table S1.** Frequency of unique chemovar index codes. **Table S2.** Descriptive statistics for side effects.

## Data Availability

The data is available upon request and approval from MoreBetter, Ltd.
