# Supplementary material for: Systematic combinations of major cannabinoid and terpene contents in Cannabis flower and patient outcomes: a proof-of-concept assessment of the Vigil Index of Cannabis Chemovars
Source: J Cannabis Res. 2023 Feb 8;5:4. doi: 10.1186/s42238-022-00170-9 (PMC9906924; doi:10.1186/s42238-022-00170-9)
Supplement: Supplementary file 1 — Additional file 1: Table S1. Frequency of unique chemovar index codes. Table S2. Descriptive statistics for side effects. [file 42238_2022_170_MOESM1_ESM.docx]

Supplementary Materials

**This file includes:**

Tables S1 and S2

**Supplemental Table S1:** Frequency of unique chemovar index codes in the VICC

| VICC Code Frequency Percent | | |
| --- | --- | --- |
| A-70 | 4 | .1 |
| A-72 | 4 | .1 |
| A/B/MA/B/M44 | 47 | .7 |
| A/MA/M21 | 11 | .2 |
| A+-61 | 1 | .0 |
| A++/B++/M++/U++A++/B++/M++/U++ | 7 | .1 |
| A++/C++/L++A++/C++/L++52 | 15 | .2 |
| A++/C++/M++/O++A++/C++/M++/O++ | 8 | .1 |
| A++/M++A++/M++50 | 8 | .1 |
| A+++-60 | 7 | .1 |
| A+++B++70 | 1 | .0 |
| A+++B+80 | 4 | .1 |
| A+++B61 | 4 | .1 |
| A+++C+++60 | 5 | .1 |
| A+++C15 | 1 | .0 |
| A+++M+++51 | 3 | .0 |
| A+++R+++70 | 4 | .1 |
| A++L+61 | 9 | .1 |
| A+B50 | 3 | .0 |
| A+C/N/L61 | 40 | .6 |
| A+M+60 | 1 | .0 |
| A+M+61 | 9 | .1 |
| A+M61 | 24 | .4 |
| AC50 | 8 | .1 |
| AL23 | 8 | .1 |
| AL26 | 6 | .1 |
| AL50 | 12 | .2 |
| AM45 | 15 | .2 |
| AM51 | 15 | .2 |
| AM60 | 8 | .1 |
| AM71 | 4 | .1 |
| B/C/R/N/L/M41 | 2 | .0 |
| B++-71 | 1 | .0 |
| B++/C++/M++B++/C++/M++52 | 7 | .1 |
| B++/L++/M++B++/L++/M++62 | 24 | .4 |
| B+++/M+++B+++/M+++51 | 11 | .2 |
| B+++B+++60 | 4 | .1 |
| B+++C++44 | 4 | .1 |
| B+++M+++50 | 9 | .1 |
| B+++M+++51 | 3 | .0 |
| B+A+61 | 2 | .0 |
| B+L60 | 8 | .1 |
| B+L82 | 5 | .1 |
| B+M+70 | 2 | .0 |
| B+M10 | 5 | .1 |
| B+M60 | 3 | .0 |
| BC/R80 | 1 | .0 |
| BM70 | 4 | .1 |
| C-35 | 6 | .1 |
| C/LC/L60 | 29 | .5 |
| C/LM51 | 8 | .1 |
| C/LM60 | 3 | .0 |
| C/LM70 | 8 | .1 |
| C/M-61 | 1 | .0 |
| C/N/L/MC/N/L/M80 | 20 | .3 |
| C+/L+M70 | 4 | .1 |
| C+/M+C+/M+60 | 9 | .1 |
| C+/N+C+/N+61 | 8 | .1 |
| C++-70 | 1 | .0 |
| C++/H++C++/H++70 | 16 | .3 |
| C++/L++/M++-61 | 1 | .0 |
| C++/L++/M++C++/L++/M++52 | 8 | .1 |
| C++/M++/T++C++/M++/T++12 | 27 | .4 |
| C++/N++/L++/M++-80 | 3 | .0 |
| C+++-60 | 4 | .1 |
| C+++-61 | 4 | .1 |
| C+++-70 | 2 | .0 |
| C+++/M+++-80 | 1 | .0 |
| C+++C+++60 | 7 | .1 |
| C+++H++60 | 4 | .1 |
| C+++H++70 | 3 | .0 |
| C+++J60 | 1 | .0 |
| C+++M+++61 | 1 | .0 |
| C+++O+++61 | 3 | .0 |
| C++H+80 | 1 | .0 |
| C++L++33 | 3 | .0 |
| C++L60 | 3 | .0 |
| C++M+60 | 6 | .1 |
| C++M+70 | 15 | .2 |
| C++M+80 | 1 | .0 |
| C++N50 | 2 | .0 |
| C+H10 | 3 | .0 |
| C+H45 | 16 | .3 |
| C+H61 | 2 | .0 |
| C+H70 | 26 | .4 |
| C+H71 | 12 | .2 |
| C+H81 | 6 | .1 |
| C+L+50 | 6 | .1 |
| C+L+51 | 5 | .1 |
| C+L+70 | 17 | .3 |
| C+L+71 | 8 | .1 |
| C+L+80 | 4 | .1 |
| C+L50 | 12 | .2 |
| C+L60 | 11 | .2 |
| C+L61 | 11 | .2 |
| C+L70 | 22 | .3 |
| C+L71 | 6 | .1 |
| C+L80 | 6 | .1 |
| C+L81 | 8 | .1 |
| C+M+70 | 8 | .1 |
| C+M+71 | 6 | .1 |
| C+M+80 | 3 | .0 |
| C+M50 | 11 | .2 |
| C+M60 | 5 | .1 |
| C+M61 | 1 | .0 |
| C+M81 | 8 | .1 |
| C+N70 | 11 | .2 |
| C+T62 | 39 | .6 |
| CA15 | 8 | .1 |
| CH50 | 6 | .1 |
| CH61 | 21 | .3 |
| CL10 | 9 | .1 |
| CL36 | 5 | .1 |
| CL50 | 3 | .0 |
| CL60 | 21 | .3 |
| CL61 | 5 | .1 |
| CL70 | 24 | .4 |
| CL71 | 8 | .1 |
| CL80 | 4 | .1 |
| CL81 | 44 | .7 |
| CM38 | 7 | .1 |
| CM50 | 25 | .4 |
| CM60 | 30 | .5 |
| CM61 | 9 | .1 |
| CM70 | 21 | .3 |
| CN70 | 3 | .0 |
| CO51 | 13 | .2 |
| CT51 | 128 | 2.0 |
| CT60 | 9 | .1 |
| DE46 | 2 | .0 |
| E-11 | 2 | .0 |
| E+B+70 | 6 | .1 |
| E+L60 | 2 | .0 |
| F+++-51 | 4 | .1 |
| F+++-60 | 1 | .0 |
| G+M+50 | 1 | .0 |
| G+M+80 | 4 | .1 |
| H++-52 | 1 | .0 |
| H+++-52 | 1 | .0 |
| H+++L+++50 | 1 | .0 |
| H+++M+++50 | 1 | .0 |
| H+++M+++81 | 2 | .0 |
| H+++M++61 | 8 | .1 |
| H+++N++60 | 3 | .0 |
| H+C+34 | 2 | .0 |
| H+C34 | 4 | .1 |
| H+L+60 | 8 | .1 |
| J+++-60 | 8 | .1 |
| J+++-70 | 4 | .1 |
| J+++-71 | 4 | .1 |
| J+N+62 | 3 | .0 |
| L-60 | 5 | .1 |
| L/MC60 | 10 | .2 |
| L/ML/M70 | 29 | .5 |
| L+++-70 | 1 | .0 |
| L+++/T+++L+++/T+++61 | 1 | .0 |
| L+++C+++60 | 3 | .0 |
| L+++C+++63 | 3 | .0 |
| L+++M+++61 | 5 | .1 |
| L+++M++50 | 12 | .2 |
| L+++M++70 | 1 | .0 |
| L+++N++60 | 13 | .2 |
| L+++R+++60 | 8 | .1 |
| L++C++/O++80 | 1 | .0 |
| L++C++61 | 5 | .1 |
| L++C+60 | 12 | .2 |
| L++C+70 | 4 | .1 |
| L++C+71 | 8 | .1 |
| L++C81 | 4 | .1 |
| L++M+51 | 7 | .1 |
| L++M+60 | 5 | .1 |
| L+A/C70 | 14 | .2 |
| L+B/N81 | 1 | .0 |
| L+B61 | 8 | .1 |
| L+C/M60 | 2 | .0 |
| L+C/M81 | 39 | .6 |
| L+C+70 | 12 | .2 |
| L+C+80 | 78 | 1.2 |
| L+C10 | 8 | .1 |
| L+C51 | 129 | 2.0 |
| L+C60 | 20 | .3 |
| L+C61 | 2 | .0 |
| L+C70 | 22 | .3 |
| L+C80 | 1 | .0 |
| L+C81 | 6 | .1 |
| L+M/O70 | 12 | .2 |
| L+M+70 | 13 | .2 |
| L+M+80 | 2 | .0 |
| L+M+81 | 11 | .2 |
| L+M10 | 7 | .1 |
| L+M21 | 6 | .1 |
| L+M50 | 17 | .3 |
| L+M61 | 10 | .2 |
| L+M71 | 8 | .1 |
| L+N45 | 40 | .6 |
| L+N70 | 5 | .1 |
| L+N80 | 2 | .0 |
| L+N81 | 3 | .0 |
| LA51 | 3 | .0 |
| LA84 | 1 | .0 |
| LB/N81 | 5 | .1 |
| LC/E61 | 8 | .1 |
| LC/M70 | 10 | .2 |
| LC10 | 7 | .1 |
| LC50 | 17 | .3 |
| LC51 | 16 | .3 |
| LC60 | 26 | .4 |
| LC70 | 26 | .4 |
| LC71 | 3 | .0 |
| LC80 | 18 | .3 |
| LC81 | 1 | .0 |
| LM/O70 | 6 | .1 |
| LM40 | 14 | .2 |
| LM50 | 15 | .2 |
| LM51 | 2 | .0 |
| LM60 | 231 | 3.7 |
| LM61 | 9 | .1 |
| LM70 | 23 | .4 |
| LN60 | 24 | .4 |
| LN70 | 4 | .1 |
| LR70 | 9 | .1 |
| M-14 | 17 | .3 |
| M-34 | 1 | .0 |
| M-61 | 2 | .0 |
| M-70 | 2 | .0 |
| M/TM/T44 | 91 | 1.4 |
| M+-60 | 13 | .2 |
| M+-71 | 1 | .0 |
| M++-15 | 1 | .0 |
| M++-60 | 15 | .2 |
| M++-80 | 15 | .2 |
| M++/T++C/L51 | 7 | .1 |
| M+++-80 | 5 | .1 |
| M+++A+++51 | 11 | .2 |
| M+++A+++61 | 1 | .0 |
| M+++A++61 | 1 | .0 |
| M+++A+70 | 4 | .1 |
| M+++A60 | 11 | .2 |
| M+++L+++60 | 5 | .1 |
| M+++L++60 | 2 | .0 |
| M+++L50 | 4 | .1 |
| M+++L60 | 4 | .1 |
| M+++L70 | 7 | .1 |
| M+++M+++60 | 8 | .1 |
| M+++N61 | 12 | .2 |
| M+++O+++70 | 3 | .0 |
| M+++O70 | 6 | .1 |
| M+++O80 | 12 | .2 |
| M++A/O70 | 7 | .1 |
| M++A++70 | 1 | .0 |
| M++A+33 | 7 | .1 |
| M++A10 | 4 | .1 |
| M++A34 | 11 | .2 |
| M++A35 | 16 | .3 |
| M++A45 | 1 | .0 |
| M++A50 | 42 | .7 |
| M++A60 | 16 | .3 |
| M++A76 | 12 | .2 |
| M++A80 | 8 | .1 |
| M++C/L81 | 1 | .0 |
| M++C+20 | 2 | .0 |
| M++C+70 | 17 | .3 |
| M++C+80 | 25 | .4 |
| M++C50 | 7 | .1 |
| M++C56 | 6 | .1 |
| M++C60 | 7 | .1 |
| M++C70 | 9 | .1 |
| M++C80 | 11 | .2 |
| M++L+60 | 4 | .1 |
| M++L+70 | 5 | .1 |
| M++L+71 | 19 | .3 |
| M++L+80 | 1 | .0 |
| M++L+81 | 7 | .1 |
| M++L60 | 10 | .2 |
| M++L61 | 8 | .1 |
| M++L62 | 66 | 1.0 |
| M++L70 | 5 | .1 |
| M++L71 | 4 | .1 |
| M++N+81 | 5 | .1 |
| M++O+80 | 8 | .1 |
| M++O10 | 3 | .0 |
| M++O60 | 26 | .4 |
| M++R70 | 16 | .3 |
| M+A/B/C/N/L34 | 7 | .1 |
| M+A/C/L70 | 1 | .0 |
| M+A/H/L15 | 40 | .6 |
| M+A/L15 | 1 | .0 |
| M+A/N50 | 2 | .0 |
| M+A/R60 | 1 | .0 |
| M+A+60 | 10 | .2 |
| M+A+70 | 8 | .1 |
| M+A+80 | 16 | .3 |
| M+A15 | 6 | .1 |
| M+A33 | 27 | .4 |
| M+A34 | 11 | .2 |
| M+A40 | 91 | 1.4 |
| M+A50 | 212 | 3.4 |
| M+A60 | 22 | .3 |
| M+AR60 | 2 | .0 |
| M+C+50 | 2 | .0 |
| M+C+60 | 1 | .0 |
| M+C+70 | 7 | .1 |
| M+C+80 | 14 | .2 |
| M+C+81 | 10 | .2 |
| M+C10 | 7 | .1 |
| M+C16 | 17 | .3 |
| M+C33 | 5 | .1 |
| M+C50 | 77 | 1.2 |
| M+C51 | 98 | 1.6 |
| M+C60 | 38 | .6 |
| M+C61 | 11 | .2 |
| M+C70 | 22 | .3 |
| M+C71 | 17 | .3 |
| M+C80 | 13 | .2 |
| M+D81 | 1 | .0 |
| M+F+70 | 9 | .1 |
| M+J35 | 3 | .0 |
| M+L+50 | 9 | .1 |
| M+L+60 | 17 | .3 |
| M+L+70 | 4 | .1 |
| M+L+80 | 1 | .0 |
| M+L+81 | 21 | .3 |
| M+L10 | 6 | .1 |
| M+L35 | 15 | .2 |
| M+L50 | 10 | .2 |
| M+L60 | 64 | 1.0 |
| M+L70 | 19 | .3 |
| M+L71 | 2 | .0 |
| M+L80 | 12 | .2 |
| M+N33 | 5 | .1 |
| M+N34 | 4 | .1 |
| M+N54 | 3 | .0 |
| M+N60 | 1 | .0 |
| M+N61 | 1 | .0 |
| M+O41 | 6 | .1 |
| M+O70 | 8 | .1 |
| M+R10 | 8 | .1 |
| M+R50 | 5 | .1 |
| M+R60 | 1 | .0 |
| M+T+61 | 9 | .1 |
| M+T15 | 8 | .1 |
| M+T40 | 1 | .0 |
| M+T50 | 7 | .1 |
| M+T60 | 1 | .0 |
| M+T61 | 20 | .3 |
| MA/L/O70 | 5 | .1 |
| MA10 | 1 | .0 |
| MA16 | 1 | .0 |
| MA26 | 1 | .0 |
| MA50 | 127 | 2.0 |
| MA51 | 1 | .0 |
| MA61 | 11 | .2 |
| MA72 | 8 | .1 |
| MB50 | 5 | .1 |
| MB51 | 2 | .0 |
| MB71 | 1 | .0 |
| MC/L16 | 4 | .1 |
| MC14 | 1 | .0 |
| MC28 | 1 | .0 |
| MC35 | 2 | .0 |
| MC40 | 6 | .1 |
| MC43 | 4 | .1 |
| MC50 | 38 | .6 |
| MC51 | 5 | .1 |
| MC54 | 11 | .2 |
| MC60 | 17 | .3 |
| MC61 | 223 | 3.5 |
| MC62 | 211 | 3.3 |
| MC70 | 9 | .1 |
| MC72 | 5 | .1 |
| MC80 | 22 | .3 |
| MH50 | 5 | .1 |
| ML50 | 17 | .3 |
| ML60 | 70 | 1.1 |
| ML70 | 23 | .4 |
| MN64 | 4 | .1 |
| MO/T50 | 8 | .1 |
| MO10 | 1 | .0 |
| MO71 | 8 | .1 |
| MT40 | 1 | .0 |
| MT50 | 81 | 1.3 |
| MT55 | 2 | .0 |
| MT60 | 15 | .2 |
| MT61 | 21 | .3 |
| N-51 | 1 | .0 |
| N++/L++/E++N++/L++/E++51 | 8 | .1 |
| N++/L++/M++N++/L++/M++70 | 7 | .1 |
| N+++-60 | 8 | .1 |
| N+++-61 | 1 | .0 |
| N+++-71 | 4 | .1 |
| N+++C+++81 | 4 | .1 |
| N+++L+++60 | 3 | .0 |
| N+++M+++71 | 4 | .1 |
| N++L++43 | 5 | .1 |
| N++N++52 | 8 | .1 |
| N+L70 | 5 | .1 |
| N+M+71 | 1 | .0 |
| NB71 | 7 | .1 |
| NC10 | 12 | .2 |
| NC50 | 3 | .0 |
| O+++-61 | 4 | .1 |
| O+++L++61 | 7 | .1 |
| O++M++35 | 16 | .3 |
| O++M++60 | 2 | .0 |
| O++M+70 | 12 | .2 |
| O+L+80 | 3 | .0 |
| O+M+70 | 13 | .2 |
| OM71 | 8 | .1 |
| P++-47 | 3 | .0 |
| P++-61 | 3 | .0 |
| P+++-51 | 1 | .0 |
| P+M+60 | 4 | .1 |
| R++/L++/M++R++/L++/M++52 | 12 | .2 |
| R++/M++R++/M++60 | 2 | .0 |
| R++/O++/T++R++/O++/T++52 | 12 | .2 |
| R+++/M+++/T+++R+++/M+++/T+++61 | 9 | .1 |
| R+++R+++80 | 7 | .1 |
| R+B+51 | 10 | .2 |
| R+C60 | 87 | 1.4 |
| R+H80 | 1 | .0 |
| R+L60 | 6 | .1 |
| R+L71 | 2 | .0 |
| RB51 | 7 | .1 |
| RH41 | 2 | .0 |
| RH60 | 10 | .2 |
| RL50 | 15 | .2 |
| RL60 | 5 | .1 |
| RM60 | 11 | .2 |
| RM70 | 9 | .1 |
| RN71 | 2 | .0 |
| RR80 | 8 | .1 |
| T+++-73 | 4 | .1 |
| T+++M70 | 40 | .6 |
| T++C10 | 4 | .1 |
| T++C60 | 10 | .2 |
| T++C70 | 19 | .3 |
| T++L50 | 5 | .1 |
| T++L70 | 149 | 2.4 |
| T++M+60 | 14 | .2 |
| T++M+61 | 9 | .1 |
| T++M+70 | 4 | .1 |
| T++M+80 | 1 | .0 |
| T++M60 | 2 | .0 |
| T++M61 | 19 | .3 |
| T++M70 | 9 | .1 |
| T+C51 | 8 | .1 |
| T+C70 | 11 | .2 |
| T+L60 | 222 | 3.5 |
| T+M/O82 | 6 | .1 |
| T+M33 | 5 | .1 |
| T+M34 | 3 | .0 |
| T+M50 | 1 | .0 |
| T+M60 | 17 | .3 |
| T+M61 | 10 | .2 |
| T+M70 | 12 | .2 |
| T+O+61 | 1 | .0 |
| T+O50 | 17 | .3 |
| T+O60 | 6 | .1 |
| TA/N40 | 2 | .0 |
| TA50 | 5 | .1 |
| TA60 | 3 | .0 |
| TC/L/M/O80 | 7 | .1 |
| TC60 | 9 | .1 |
| TC61 | 3 | .0 |
| TH62 | 11 | .2 |
| TL50 | 8 | .1 |
| TL60 | 5 | .1 |
| TM33 | 5 | .1 |
| TM40 | 15 | .2 |
| TM51 | 15 | .2 |
| TM60 | 8 | .1 |
| TM61 | 29 | .5 |
| TM71 | 11 | .2 |
| TM73 | 5 | .1 |
| TO50 | 19 | .3 |
| TR50 | 1 | .0 |
| TR71 | 3 | .0 |
| V+C60 | 5 | .1 |
| Total | 6309 | 100.0 |

Note. The first two places in the four unit Vigil Index of Cannabis Chemovars (VICC) are the alphabetic symbols for 20 terpenes, with the highest concentrated terpene(s) in the first place and the terpene(s) with the 2^nd^ highest concentration in the second place. The relative magnitudes of each units concentration levels are indicated with the presence of superscript(s) “^+^” following the alphabetic symbol: (no superscript) = 0.01 to 0.49%/dry wt; “^+^” = 0.50% to 0.99%/dry wt; “^++^” = 1.00% to 1.99%/dry wt; and “^+++^” = 2.00% to 3.00%/dry wt. The “-” indicates the absence of a 2^nd^ identified terpene and a “/” between terpenes in the 1^st^ and 2^nd^ places indicate exactly matched concentration levels. The third place unit indicates THC levels: 1= 0-.9%; 2 = 1-4.9%; 3 = 5-9.9%; 4 = 10-14.9%; 5 = 15-19.9%; 6 = 20-24.9%; 7 = 25-29.9%; 8 = 30-35%., and the fourth place unit indicates CBD levels: 0 = 0%; 1 = .01-.9%; 2 = 1-4.9%; 3 = 5-9.9%; 4 = 10-14.9%; 5 = 15-19.9%; 6 = 20-24.9%; 7 = 25-29.9%; 8 = 30-35%.

**Supplemental Table 2:** Descriptive statistics for side effects

| **Side Effect** | **N sessions** | **% Sessions Reporting** | **Category** |
| --- | --- | --- | --- |
| Anxious | 210 | 3.3% | Negative |
| Clumsy | 509 | 8.1% | Negative |
| Confused | 126 | 2.0% | Negative |
| Coughing | 898 | 14.2% | Negative |
| Dizzy | 280 | 4.4% | Negative |
| Dry Mouth | 2562 | 40.6% | Negative |
| Foggy | 537 | 8.5% | Negative |
| Forgetful | 537 | 8.5% | Negative |
| Headache | 251 | 4.0% | Negative |
| Irritable | 243 | 3.9% | Negative |
| Nausea | 86 | 1.4% | Negative |
| Paranoid | 108 | 1.7% | Negative |
| Rapid Pulse | 193 | 3.1% | Negative |
| Red Eyes | 1692 | 26.8% | Negative |
| Restless | 511 | 8.1% | Negative |
| Scattered | 893 | 14.2% | Negative |
| Unmotivated | 477 | 7.6% | Negative |
| Active | 532 | 8.4% | Positive |
| Chill | 3979 | 63.1% | Positive |
| Clear | 1045 | 16.6% | Positive |
| Comfy | 1826 | 28.9% | Positive |
| Creative | 709 | 11.2% | Positive |
| Dreamy | 2319 | 36.8% | Positive |
| Energetic | 697 | 11.0% | Positive |
| Focused | 1050 | 16.6% | Positive |
| Frisky | 420 | 6.7% | Positive |
| Grateful | 1419 | 22.5% | Positive |
| Great | 1138 | 18.0% | Positive |
| Happy | 1688 | 26.8% | Positive |
| Light | 1807 | 28.6% | Positive |
| Optimistic | 1673 | 26.5% | Positive |
| Peaceful | 2776 | 44.0% | Positive |
| Productive | 515 | 8.2% | Positive |
| Reflective | 1235 | 19.6% | Positive |
| Relaxed | 3543 | 56.2% | Positive |
| Tuned | 958 | 15.2% | Positive |
| Couchlocked | 699 | 11.1% | Context-Specific |
| Distracted | 536 | 8.5% | Context-Specific |
| High | 3555 | 56.3% | Context-Specific |
| Hungry | 844 | 13.4% | Context-Specific |
| Silly | 1010 | 16.0% | Context-Specific |
| Sleepy | 1298 | 20.6% | Context-Specific |
| Talkative | 329 | 5.2% | Context-Specific |
| Thinky | 1438 | 22.8% | Context-Specific |
| Thirsty | 1471 | 23.3% | Context-Specific |
| Tingly | 2083 | 33.0% | Context-Specific |
| Visuals | 542 | 8.6% | Context-Specific |

Notes: Side effects are categorized according to their visceral experiences

under varying circumstances.
